# Association of high-sensitivity cardiac troponin T with all-cause and cardiovascular mortality in a mobile cohort of older adults aged 70 to 95 years – Results from the AugUR study

**DOI:** 10.1016/j.ajpc.2026.101520

**Published:** 2026-03-03

**Authors:** Klaus J. Stark, Martina E. Zimmermann, Janina M. Herold, Caroline Brandl, Ralph Burkhardt, Lars S. Maier, Andreas Luchner, Maria A. Heinrich, Iris M. Heid, Alexander Dietl

**Affiliations:** a Department of Genetic Epidemiology, University of Regensburg, Regensburg, Germany; b Department of Ophthalmology, University Hospital Regensburg, Regensburg, Germany; c Institute of Clinical Chemistry and Laboratory Medicine, University Hospital Regensburg, Regensburg, Germany; d Department of Internal Medicine II, University Hospital Regensburg, Regensburg, Germany; e Department of Cardiology, Hospital Barmherzige Brueder Regensburg, Regensburg, Germany

**Keywords:** Population-based study, Older adults, AugUR, Mortality, high-sensitivity cardiac troponin T, Risk factors

## Abstract

**Background:**

It remains unclear whether high-sensitivity cardiac troponin T (hsTnT) independently predicts mortality in older adults.

**Objective:**

To estimate mortality rate and association of hsTnT with all-cause and cardiovascular death in the older, mobile population.

**Methods:**

This analysis was conducted in 2055 subjects of the AugUR-study, a prospective population-based cohort study in individuals aged 70 years and older with up to 10-year mortality follow-up. We used Cox proportional hazard models to estimate hazard ratios (HR) and 95% confidence intervals (CI) for association of hsTnT with all-cause, cardiovascular, non-cardiovascular and premature mortality risk. Association results of hsTnT with mortality were reported for tertiles and per 1 standard deviation (SD) of log-transformed values.

**Results:**

Participants were 70 to 95 years old with 48% men and 56% with prevalent cardiovascular disease (CVD). In a median follow-up period of 6.0 years, 385 (113 cardiovascular) deaths were recorded. For all-cause mortality, HR was 1.66 [95%CI=1.49–1.85] after adjustment for age and sex (p-value<0.001). For cardiovascular mortality, the effect was more pronounced (HR=2.17 [95%CI=1.79–2.63]; *p* < 0.001). The association between hsTnT with non-cardiovascular mortality was also significant (HR=1.61 [95%CI=1.41–1.83]; *p* < 0.001). In a full model adjusting for factors influencing mortality and hsTnT levels, in participants with prevalent CVD, hsTnT showed still association with cardiovascular mortality (HR=1.65 [95%CI=1.30–2.10]; *p* < 0.001) but also with non-cardiovascular mortality (HR=1.32 [95%CI=1.10–1.58]; *p* < 0.01).

**Conclusion:**

Elevated levels of hsTnT were associated with increased cardiovascular and non-cardiovascular mortality risk in an old-aged population. Measurement of hsTnT in subjects with prevalent CVD may help identify individuals at high risk for mortality.

## Graphical abstract

**Figure:**
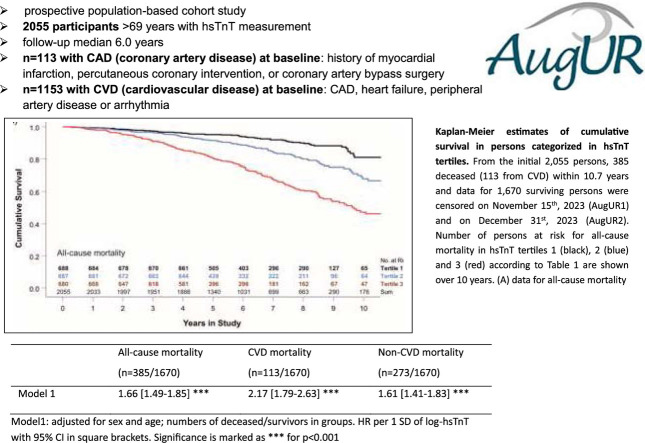

**Key messages of the article**

What is already known on this topic: Troponins are known predictors for cardiovascular and overall mortality.

What this study adds: This study focussed on a mobile cohort of older adults aged 70 to 95 years with a 10-year mortality follow-up.

How this study might affect research, practice, or policy: Troponin T, measured with a high-sensitivity assay is a predictor for mortality in older adults. This may be caused by asymptomatic myocardial damage or systemic disturbances of the vascular system and, therefore, highlights troponin T as a marker to establish protective cardiovascular therapy in this group of persons.

Alt-text: Unlabelled box dummy alt text

## 1 Introduction

In clinical practice, cardiac troponins T and I are employed for the diagnosis of acute myocardial infarction (MI) [1]. Since the upcoming of high-sensitivity assays for cardiac troponin T (hsTnT) as a marker of subclinical cardiomyocyte damage, several studies reported increased mortality with higher concentrations of hsTnT in the general population [[2], [3], [4]]. In old-aged but apparently healthy individuals free of signs for cardiovascular disease (CVD), hsTnT values were shown to be detectable and were in a substantial proportion of subjects higher than the recommended rule-out cut-off value of 14 ng/L for MI [5]. Some factors like age, sex, hypertension or diabetes were described to be associated with increased hsTnT concentrations over time [6]. Interestingly, other clinical manifestations beyond MI like atrial fibrillation, heart failure, tachycardiomyopathy and myocarditis were found to be associated with elevated cardiac troponin levels in the blood stream [[7], [8], [9], [10]]. The advantage of high-sensitivity assays for cardiac troponins is that low but measurable values below the clinical rule-out threshold may provide prognostic benefit for heart failure and CVD but are not used in clinical practice[11]. Both cardiac troponins T and I were modestly correlated in the general population and provided distinct mortality risk association [3,12]. For hsTnT it was shown that association with all-cause mortality was stronger than for hsTnI [3] and that hsTnT and not hsTnI was associated with non-cardiovascular mortality [4]. In a meta-analysis, association with fatal CVD was stronger for hsTnT rather than for hsTnI [13].

The question remains whether hsTnT is a predictor for mortality in old-aged individuals, even after exclusion of competing risk and influencing factors on hsTnT measurement.

Here, we aimed to analyse the association between serum measurements of hsTnT and all-cause as well as cardiovascular and non-cardiovascular mortality in 2055 participants of the German AugUR study (Altersbezogene Untersuchungen zur Gesundheit der Universität Regensburg) with individuals aged 70 years and older.

## 2 Methods

### 2.1 AugUR cohort study

The German AugUR study is a prospective study of the general population of older adults in and around the city of Regensburg, Bavaria. AugUR was established as a research platform to estimate the prevalence and incidence of chronic conditions and to understand associated risk factors for chronic health disabilities and mortality in older adults.

Details on the study were published earlier [[14], [15], [16], [17]]. In brief, 2449 participants aged 70 to 95 years were included in the study between 2013 and 2019 in two recruitment parts with identical study programme (AugUR1: March 2013 to November 2015, *n* = 1133; AugUR2: May 2017 to November 2019, *n* = 1316). A scheme of recruitment and response proportion is summarized in **Supplementary Figure 1**. A total of 2055 participants had values for serum hsTnT measurement and no missing data for all examined parameters and were included in the analyses.

We consider the study participants physically mobile and without major cognitive impairments, since they have had to visit the study centre at the University Hospital Regensburg and actively take part at the study program.

The AugUR study was approved by the **Ethics** Committee of the University of Regensburg, Germany (vote 12–101–0258). The study complies with the 1964 Helsinki declaration and its later amendments. All participants provided informed written consent.

### 2.2 AugUR study program

Medical **examinations** at the study centre included height, weight, hip and waist circumference, hand grip strength, blood pressure and pulse frequency.

The baseline **questionnaire** conducted as an in-person interview included information on general chronic diseases, intake of medication, socio-demographic characteristics such as sex, age, living status and education level. Data on medical history were collected via self-report using the question “Has a physician ever diagnosed one of the following conditions?”. Participants were asked if they had ever been diagnosed with hypertension, diabetes, stroke, heart failure (HF; asked as ‘heart weakness’), or peripheral artery disease (PAD; self-report of diagnosis or arterial operation of the limbs). Additionally, they were asked about any history of myocardial infarction, percutaneous coronary intervention, or coronary artery bypass surgery; **coronary artery disease (CAD)** was defined if at least one of these three conditions was reported. **Cardiovascular disease (CVD)** was defined as CAD or stroke or HF or PAD or arrhythmia (defined as irregular pulse frequency at the study centre examination). With the exception of pulse measurements, all diseases are recorded based on self-reported information provided by the study participants. In an analysis conducted as part of our study, in which we compared the self-reported information with the records kept by the treating physicians, we were able to show that there was a very high degree of consistency for conditions such as myocardial infarction [15]. **Hypertension** was defined as blood pressure ≥ 140/90 mmHg or if the individual reported a prior hypertension diagnosis and antihypertensive medication intake [18]. Individuals with self-reported diabetes and/or antidiabetic medication intake were defined as having **diabetes** [19].

Collection and processing of **biosamples** were conducted following standard operation procedures as described previously [14,16]. Briefly, non-fasting blood samples were drawn in a sitting position after at least 5 min of resting. Mild venous stasis was applied for a maximum duration of 1 min. Blood was taken using a 21 G multifly needle. Immediate measurements in fresh whole blood and serum were conducted on the same day. For biobanking, blood samples and midstream urine were processed immediately and stored at −80 °C.

**Measurements in fresh samples** were conducted by an external laboratory (Synlab, Regensburg, Germany). **HbA1c** was measured from EDTA-anticoagulated whole blood by applying ion-exchange high-performance liquid chromatography on a Bio-Rad Variant II Turbo, applying the Variant II Turbo HbA1c Kit 2.0 (Bio-Rad, Munich, Germany), and given as [ %]. **LDL-, HDL-, total cholesterol and triglycerides** were quantified as [mg/dl] from serum on a Beckman AU 5400 analyser using enzymatic tests OSR6183, OSR6187, OSR6116, and OSR60118, respectively (Beckman Coulter, Krefeld, Germany). Serum high-sensitivity C-reactive protein (**CRP**) was measured on a Beckman AU 5400 analyser using the turbidimetric immunological test OSR 6199 and quantified as [mg/l].

**Laboratory analyses from biobanked samples** were performed in compliance with the “Guidelines of the German Medical Association for Quality Assurance of Medical Laboratory Tests” (RiLiBäK) at the Central Laboratory of the University Hospital Regensburg, which is accredited in accordance with the standard DIN EN ISO 15,189. Measurements for **high-sensitivity troponin T (hsTnT)** were conducted in stored serum samples on Roche cobas e411 and e801 (Roche Diagnostics, Mannheim, Germany) with Elecsys Troponin T high-sensitive assay and reported as [ng/l]. For 41 hsTnT values on the lower detection limit of 3 ng/L (limit of blank), data were winsorised to 3.0 ng/L. Therefore, in our study group 98 % had detectable hsTnT values. The upper detection limit of the Elecsys hsTnT assay was 10,000 ng/L; the highest measured value in AugUR study participants was 421.3 ng/L. Four extreme values above 100 ng/L (421.3, 156.0, 107.1, and 102.8, respectively) were winsorised to 100 ng/L for better comparison of associations per unit of hsTnT. We modelled hsTnT as both a continuous and categorial parameter. For the continuous analyses, values for hsTnT were natural logarithm (log)-transformed and normalized based on the SD from the log-distribution; the SD-normalized range was 5.85 units. Hazard ratios per SD were reported for better comparability because variance of hsTnT values per group was slightly different. In the whole data set, one SD per log-transformed hsTnT corresponded approximately to 1.82 ng/L on the original scale. Additionally, effects per SD are better comparable with results from other studies. For the categorial analyses, individuals were classified according to the tertiles of the hsTnT distribution. **Creatinine from serum and midstream urine** was enzymatically measured in individuals recruited in the years 2013–2015 (AugUR1, *n* = 1129) on a Siemens Dimension Vista 1500 (assay ECREA, Siemens Healthcare, Erlangen, Germany) or in those recruited from 2017 to 2019 (AugUR2, *n* = 1291) on a Roche cobas e801 (assay CREP2, Roche, Mannheim, Germany) and reported as [mg/dl]. **Serum cystatin C** was measured with immunoassays for AugUR1 on a Siemens Dimension Vista 1500 (assay CYSC) or for AugUR2 on a Roche cobas e801 (assay CYSC2) and reported as [mg/dl]. **Urine albumin** was measured with immunoassays for AugUR1 on a Siemens Dimension Vista 1500 (assay MALB) or for AugUR2 on a Roche cobas e801 (assay ALBT2) and reported as [mg/l]. Comparability of methods for creatinine, cystatin C, and albumin was assessed following Clinical & Laboratory Standards Institute (CLSI) guidelines. Urine albumin from Siemens assay was calibrated with the formula “−2.94 + 0.89*Siemens” to match with the Roche assay. **Estimated glomerular filtration rate (eGFR [ml/min/1.73m²])** was derived from serum creatinine and cystatin C levels measured via enzymatic assay using the 2021 Chronic Kidney Disease Epidemiology Collaboration (CKD-Epi) combined creatinine and cystatin C equation (eGFR_crea-cys_) [20]. Definition for chronic kidney disease (CKD) at eGFR < 60 mL/min/1.73 m² corresponds to current KDIGO (Kidney Disease: Improving Global Outcomes) guidelines [21]. **Urinary creatinine-to-albumin ratio (UACR [mg/g])** was calculated from measurement of creatinine and albumin in urine. According to KDIGO guidelines [21], no albuminuria was defined as UACR < 30 mg/g, microalbuminuria as 30 mg/*g* ≤ UACR ≤ 300 mg/g and macroalbuminuria as UACR > 300 mg/g.

### 2.3 Mortality survey and causes of death in the AugUR study

For all AugUR study participants central registers were queried in November/December 2023 to determine individual mortality. The last date of mortality survey was December 31st, 2023. Observation time was censored for survivors from AugUR1 on November 15th, 2023, and to those from AugUR2 on December 31st, 2023, resulting in a follow-up period of up to 10.7 years. Based on death certificates, causes of death were classified as ‘cardiovascular’ if they included International Statistical Classification of Diseases, 10th Revision codes I00-I99. For non-cardiovascular causes of death, a strict review of death certificates was performed to exclude any cardiovascular reasons. In cases of doubt, the cause of death was classified as “unknown”. For competing risk analysis, non-cardiovascular causes of death were handled as a competitive outcome for cardiovascular death and vice versa. Mortality rate in the AugUR study was calculated and compared to German death Tables 2019/2021 (Supplementary Table 1).

### 2.4 Statistical analysis

Data management and statistical analyses were performed using SAS 9.4 software (SAS Institute Inc., Cary, NC, USA) and IBM SPSS Statistics for Windows, Version 29.0.1.1 (IBM Corp., Armonk, NY, USA). Kaplan-Meier curves and Cox proportional hazard survival regressions were performed to model the time to event data. 95 % confidence intervals (95 % CI) for hazard ratios (HR) were calculated as $e^{\left(b\pm 1.96*SE\right)}$. For testing of differences between two HRs the Wald test $Z=\frac{\ln(HR1)-\text{ln}(HR2)}{\sqrt{SE1^{2}+SE2^{2}}}$ was applied und one-sided p_Diff_ was reported. Competing risk regression was performed using the SPSS R extension package cmprsk in version 1.1.1. Two-sided p-values for comparison between two groups were derived from T-test for normal distributed continuous variables, from Mann-Whitney-*U test* for not normal distributed continuous variables, and from Chi²-test for nominal variables. Significance was marked as * for *p* < 0.05, ** for *p* < 0.01, *** for *p* < 0.001, n.s. not significant (*p* ≥ 0.05).

To identify competing factors on mortality, Cox proportional hazard survival regression was applied with −2-log-liklehood-ratio forward selection with baseline variables (Supplementary Table 2). In addition, linear regression with forward selection was used to determine factors influencing baseline hsTnT values (Supplementary Table 3). Significant variables were used for adjusting in the models. Results were reported from unadjusted model (only hsTnT), model 1 (adding age and sex), model 2 (adding eGFR and UACR), model 3 (adding diabetes, BMI, physical activity, as well as intake of antihypertensive drugs, lipid-lowering medication and high-ceiling diuretics), and model 3 with prevalent CAD and CVD, respectively.

## 3 Results

### 3.1 Characteristics of individuals in the AugUR study population

From the 2055 individuals at baseline, 385 deceased during a median follow-up time of 6.0 years (interquartile range, 4.6 - 8.3; mean follow-up 6.3 ± 2.3 years; maximum 10.7 years). In comparison to the expected mortality rate in the general German population, observed 5-year mortality rate in AugUR study participants matched for age groups and sex was 52.6 % (**Supplementary Table 1**). Overall, all-cause mortality rate in men is higher than in women (63.6 %). CVD mortality was recorded for 113 individuals (29.4 % of death causes; 65.5 % men). A total of 324 participants reported CAD at baseline (15.8 %), and 1153 participants reported CVD at baseline (56.1 %). The characteristics of all AugUR participants at baseline and for mortality are shown in Table 1.

**Table 1:** Baseline characteristics and mortality data of AugUR participants overall and by **hsTnT tertiles**.

Characteristic	Overall (*n* = 2055)	Tertile 1 (*n* = 688)	Tertile 2 (*n* = 687)	Tertile 3 (*n* = 680)
*General descriptives*				
Age [yrs]	78.3 ± 5.0	76.2 ± 3.9	78.2 ± 4.3	80.5 ± 5.5
Age range [yrs]	70.3 – 95.0	70.3 – 92.3	70.3 – 95.0	70.4 – 95.0
Sex, men	981 (47.7)	188 (27.3)	345 (50.2)	448 (65.9)
Never smoked	1129 (54.9)	411 (59.7)	383 (55.7)	335 (49.3)
Smoked within last 15 yrs	219 (10.7)	76 (11.0)	87 (12.7)	56 (8.2)
Grip strength [kg]	30.2 ± 9.8	28.4 ± 9.1	31.3 ± 10.4	31.1 ± 9.6
Physical activity≥2 hrs/week	1658 (80.7)	586 (85.2)	568 (82.7)	504 (74.1)
BMI [kg/m²]	27.7 ± 4.5	26.9 ± 4.2	27.7 ± 4.2	28.3 ± 4.8
WHR	0.95 ± 0.09	0.92 ± 0.08	0.95 ± 0.09	0.98 ± 0.09
Systolic BP [mmHg]	131.8 ± 18.0	131.3 ± 17.2	132.8 ± 17.5	131.4 ± 19.1
Diastolic BP [mmHg]	76.2 ± 10.6	77.8 ± 10.2	76.7 ± 10.3	74.0 ± 11.1
Pulse frequency [min^-1^]	69.4 ± 11.5	70.3 ± 10.8	68.6 ± 10.9	69.4 ± 12.6
*Medication*				
Antihypertensive drugs	1378 (67.1)	385 (56.0)	460 (67.0)	533 (78.4)
High-ceiling diuretics	267 (13.0)	23 (3.3)	56 (8.2)	188 (27.6)
Lipid-lowering	725 (35.3)	197 (28.6)	239 (34.8)	289 (42.5)
Antidiabetics	339 (16.5)	71 (10.3)	95 (13.8)	173 (35.4)
*Laboratory values*				
hsTnT [ng/L]	10.4 [7.2;15.3]	6.3 [5.1;7.3]	10.5 [9.4;11.9]	18.7 [15.3;25.0]
hsTnT, min – max [ng/L]	3.0 – 100	3.0 – 8.2	8.3 – 13.3	13.4 – 100
eGFR [ml/min/1.73m²]	69.3 ± 17.1	77.5 ± 13.9	70.6 ± 15.2	59.8 ± 17.1
UACR [mg/g]	10.0 [5.1;22.6]	8.4 [4.5;15.1]	8.7 [4.6;19.3]	15.0 [6.9;44.8]
LDL cholesterol [mg/dl]	141.1 ± 34.9	150.8 ± 33.7	139.8 ± 33.7	132.5 ± 34.9
HDL cholesterol [mg/dl]	61.4 ± 15.5	64.9 ± 14.6	61.1 ± 15.4	58.2 ± 15.6
Total cholesterol [mg/dl]	217.7 ± 45.9	231.4 ± 44.1	216.2 ± 44.1	205.3 ± 45.8
Triglycerides [mg/dl]	137 [99;189]	139 [101;188]	139 [99;199]	135 [95;195]
HbA1c [ %]	5.8 ± 0.7	5.7 ± 0.6	5.8 ± 0.6	5.9 ± 0.8
CRP [mg/l]	0.3 [0.3;0.4]	0.3 [0.3;0.4]	0.3 [0.3;0.4]	0.3 [0.3;0.5]
*Diseases*				
CAD	324 (15.8)	49 (7.1)	89 (13.0)	186 (27.4)
CVD	1153 (56.1)	295 (42.9)	362 (52.7)	496 (72.9)
Diabetes mellitus	434 (21.1)	92 (13.4)	134 (19.5)	208 (30.6)
Hypertension	1495 (72.7)	442 (64.2)	510 (74.2)	543 (79.9)
CKD	572 (27.8)	79 (11.5)	164 (23.9)	329 (48.4)
Microalbuminuria	360 (17.5)	81 (11.8)	100 (14.6)	179 (26.3)
Macroalbuminuria	56 (2.7)	3 (0.4)	6 (0.9)	47 (6.9)
*Mortality data*				
Follow-up time overall	6.0 [4.6;8.3]	6.3 [4.9;8.7]	5.9 [4.5;8.2]	5.6 [4.4;7.5]
Survivors	1670 (81.3)	624 (90.7)	578 (84.1)	468 (68.8)
Follow-up time	6.1 [4.8;8.5]	6.4 [5.1;8.8]	6.0 [4.7;8.4]	6.0 [4.8;8.2]
All-cause mortality	385 (18.7)	64 (9.3)	109 (15.9)	212 (31.2)
Follow-up time deceased	4.6 [2.9;6.8]	4.7 [2.7;7.4]	4.8 [3.2;7.1]	4.4 [2.8;6.2]
Deceased men	245 (63.6)	24 (37.5)	64 (58.7)	157 (74.1)
CVD mortality	113 (6.3)	12 (1.9)	27 (4.5)	74 (13.7)
Follow-up time deceased	5.2 [3.3;7.3]	6.5 [4.2;7.8]	5.6 [3.4;7.6]	4.6 [3.2;6.6]
Deceased men	74 (65.5)	1 (8.3)	17 (63.0)	56 (75.7)
Non-CVD mortality	272 (14.0)	52 (7.7)	82 (12.4)	138 (22.3)
Follow-up time deceased	4.4 [2.7;6.6]	4.2 [2.4;7.3]	4.7 [3.0;6.9]	4.3 [2.6;6.2]
Deceased men	171 (62.9)	23 (44.2)	47 (57.3)	101 (73.2)

Continuous values are means ± standard deviation if normally distributed; other continuous values are median with 25 %^th^; 75 %^th^ quartiles in square brackets; categorial variables are total numbers and percent in brackets.

BMI, body mass index; WHR, waist-hip ratio; BP blood pressure; hsTnT, high-sensitivity cardiac troponin T; eGFR, estimated glomerular filtration rate calculated using serum creatinine and cystatin C applying the CKD-Epi formula 2021[20]; UACR, urinary albumin-to-creatinine ratio; LDL, low-density lipoprotein; HDL, high-density lipoprotein; HbA1c, glycated haemoglobin A1c; CRP, C-reactive protein; CAD, coronary artery disease defined as self-reported history of myocardial infarction and/or percutaneous coronary intervention and/or coronary bypass surgery; CVD, defined as CAD or stroke or HF (self-reported heart failure, “heart weakness”) or arrhythmia (defined as measured irregular pulse at study centre) or PAD (peripheral artery disease); CKD, chronic kidney disease defined as eGFR < 60 ml/min/1.73m²; microalbuminuria defined UACR 30–300 mg/g; macroalbuminuria defined as UACR > 300 mg/g. For CVD mortality, non-CVD death was set as missing and vice versa.

**Fig. 1:**
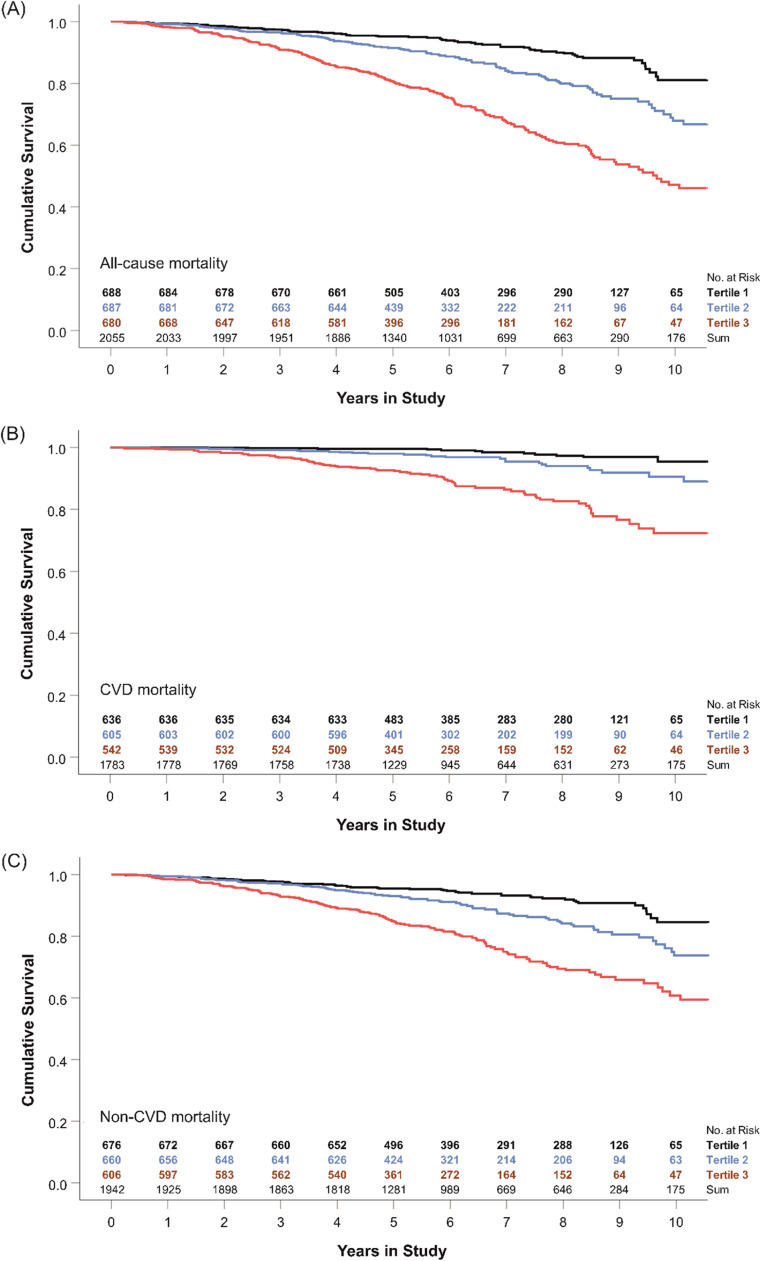
Kaplan-Meier estimates of cumulative survival in persons categorized in hsTnT tertiles. From the initial 2055 persons, 385 deceased (113 from CVD) within 10.7 years and data for 1670 surviving persons were censored on November 15th, 2023 (AugUR1) and on December 31st, 2023 (AugUR2). Number of persons at risk for all-cause mortality in hsTnT tertiles 1 (black), 2 (blue) and 3 (red) according to Table 1 are shown over 10 years. (A) data for all-cause mortality, (B) data for CVD mortality, (C) data for non-CVD mortality.

In comparison to hsTnT tertile 1, participants in tertile 2 and 3 were more likely to be older and male; were less likely to have never smoked and to be physically active; had higher BMI, WHR and medication intake; had lower cholesterol levels; showed higher prevalences in CAD, CVD, diabetes mellitus, hypertension, CKD and albuminuria, and showed higher mortality risk (Table 1). To visualize the influence of hsTnT in tertiles on mortality risk, Kaplan-Meier curves were generated for all-cause, CVD and non-CVD mortality (Fig. 1).

### 3.2 Association of hsTnT tertiles with mortality

Testing for association with CVD mortality and for the influence on hsTnT serum levels revealed that age, sex, eGFR, UACR, diabetes, BMI, physical activity as well as intake of antihypertensive drugs, lipid-lowering medication and high-ceiling diuretics should be considered for further analyses (**Supplementary Tables 2 and 3**). Prevalent CAD and CVD were analysed in separate models. To get an overview of the association between hsTnT and mortality, different models were analysed with Cox proportional hazard survival regression (Table 2). Unadjusted HRs were derived for all-cause, CVD and non-CVD mortality for categorial hsTnT tertiles. To evaluate for the stepwise influence of different variables on the effect of hsTnT tertiles on mortality, first baseline age and sex were added (model 1), followed by model 2 with kidney function (eGFR_crea-cys_ and UACR). Model 3 included additionally diabetes, BMI, physical activity as well as intake of antihypertensive drugs, lipid-lowering medication, and high-ceiling diuretics. To analyse the additional effect of CAD or CVD these disease entities were included in model3+CAD and model3+CVD, respectively.

**Table 2:** Hazard ratios from Cox proportional hazard survival regression for mortality by tertiles of hsTnT distribution.

# for mortality analyses: All-cause/CVD/non-CVD	hsTnT tertile 1 *n* = 688/636/676	hsTnT tertile 2 *n* = 687/605/660	hsTnT tertile 3 *n* = 680/542/606
All-cause mortality	64 (9.3)	109 (15.9)	212 (31.2)
Unadjusted	Reference	1.94 [1.42;2.64] ***	4.28 [3.23;5.66] ***
Model 1	Reference	1.51 [1.10;2.07] *	2.49 [1.82;3.42] ***
Model 2	Reference	1.37 [0.99;1.88] n.s.	1.72 [1.23;2.41] **
Model 3	Reference	1.40 [1.01;1.93] *	1.70 [1.21;2.39] **
Model 3+CAD	Reference	1.42 [1.03;1.96] *	1.70 [1.20;2.40] **
Model 3+CVD	Reference	1.40 [1.01;1.92] *	1.65 [1.18;2.33] **
CVD mortality	12 (1.9)	27 (4.5)	74 (13.7)
Unadjusted	Reference	2.72 [1.38;5.37] **	9.03 [4.90;16.63] ***
Model 1	Reference	2.04 [1.02;4.07] *	4.73 [2.43;9.22] ***
Model 2	Reference	1.77 [0.88;3.55] n.s.	2.84 [1.41;5.73] **
Model 3	Reference	1.82 [0.90;3.66] n.s.	2.86 [1.41;5.79] **
Model 3+CAD	Reference	1.92 [0.95;3.88] n.s.	2.98 [1.45;6.12] **
Model 3+CVD	Reference	1.82 [0.90;3.65] n.s.	2.77 [1.37;5.62] **
Non-CVD mortality	52 (7.7)	82 (12.4)	138 (22.8)
Unadjusted	Reference	1.81 [1.28;2.56] ***	3.62 [2.62;4.98] ***
Model 1	Reference	1.39 [0.97;1.99] n.s.	2.11 [1.47;3.03] ***
Model 2	Reference	1.28 [0.89;1.83] n.s.	1.49 [1.01;2.19] *
Model 3	Reference	1.30 [0.90;1.86] n.s.	1.49 [1.00;2.20] *
Model 3+CAD	Reference	1.30 [0.91;1.87] n.s.	1.48 [1.00;2.19] n.s.
Model 3+CVD	Reference	1.30 [0.90;1.86] n.s.	1.45 [0.98;2.14] n.s.

Given are HR and [95 % CI] for tertile 2 and 3, respectively, against tertile 1 as reference for all-cause, CVD and non-CVD mortality (number and percentage of deceased are given). Significance is marked as * for *p* < 0.05, ** for *p* < 0.01, *** for *p* < 0.001, n.s. not significant (*p* > 0.05). Note: one model per line with hsTnT tertiles as one categorial variable was applied. Unadjusted: hsTnT tertiles; Model 1: including age and sex; Model 2: additionally eGFR_crea-cys_ and UACR (on log-scale); Model 3: additionally diabetes, BMI, physical activity as well as intake of antihypertensive drugs, lipid-lowering medication and high-ceiling diuretics; Model 3+CAD: as model 3 with CAD; Model 3+CVD: as model 3 with CVD.

In model 3 with prevalent CAD or CVD, only for all-cause and CVD mortality significant associations could be observed. In the case of CVD mortality, the effect of hsTnT was more attenuated after CVD than for CAD. For non-CVD mortality, even tertile 3 showed no association after inclusion of CAD or CVD.

Analyses using the assay-specific 99th percentile upper reference limit (14 ng/L for hsTnT), as recommended in the Universal Definition of Myocardial Infarction and ACC/AHA guidelines[22,23], showed higher mortality risks in participants above the threshold and are presented in Supplementary Table 4.

### 3.3 Association of continuous hsTnT values with mortality

To quantify the association between hsTnT as a continuous variable and mortality, the same models as above were analysed with Cox proportional hazard survival regression with per 1 SD of log-transformed hsTnT values (Table 3).

**Table 3:** Cox regression models for association between per 1 SD of log-transformed hsTnT and mortality.

	All-cause mortality (*n* = 385/1670)	CVD mortality (*n* = 113/1670)	Non-CVD mortality (*n* = 273/1670)	Premature mortality (*n* = 80/1670)
Unadjusted	1.93 [1.77–2.11] ***	2.51 [2.14–2.94] ***	1.89 [1.69–2.12] ***	1.48 [1.19–1.84] ***
Model 1	1.66 [1.49–1.85] ***	2.17 [1.79–2.63] ***	1.61 [1.41–1.83] ***	2.07 [1.69–2.54] ***
Model 2	1.43 [1.26–1.62] ***	1.79 [1.43–2.23] ***	1.39 [1.19–1.62] ***	1.78 [1.40–2.26] ***
Model 3	1.36 [1.20–1.55] ***	1.65 [1.32–2.07] ***	1.34 [1.15–1.58] ***	1.78 [1.39–2.23] ***
Model 3+CAD	1.35 [1.19–1.53] ***	1.61 [1.29–2.00] ***	1.34 [1.14–1.56] ***	1.76 [1.37–2.25] ***
Model 3+CVD	1.34 [1.18–1.52] ***	1.62 [1.29–2.02] ***	1.32 [1.13–1.55] ***	1.75 [1.36–2.24] ***

Given are HR and [95 % CI] per 1 SD of log-hsTnT for all-cause, CVD and non-CVD mortality. Significance is marked as * for *p* < 0.05, ** for *p* < 0.01, *** for *p* < 0.001, n.s. not significant (*p* > 0.05). Unadjusted: SD-normalized log-scaled hsTnT, continuous; Model 1: including age and sex; Model 2: additionally eGFR_crea-cys_ and UACR (on log-scale); Model 3: additionally diabetes, BMI, physical activity as well as intake of antihypertensive drugs, lipid-lowering medication and high-ceiling diuretics; Model 3+CAD: as model 3 with CAD; Model 3+CVD: as model 3 with CVD.

Age- and sex-adjustment resulted in lower HRs for hsTnT effect on all-cause, CVD and non-CVD mortality. Additional adjustment for kidney function attenuated the HRs, but association between hsTnT and mortality remained highly significant (all *p* < 0.001). Comparing the unadjusted HRs with HRs from model 2 for all-cause, CVD and non-CVD mortality resulted in significant differences (p_Diff_<0.0001, p_Diff_=0.007, p_Diff_=0.0007, respectively). However, adjustment for diabetes, BMI, physical activity as well as intake of antihypertensive drugs, lipid-lowering medication, and high-ceiling diuretics as well as prevalent CAD or CVD did not substantially attenuate the association between hsTnT and death (p_Diff_>0.05). In model 3, HR per 1 SD of log-transformed hsTnT for CVD mortality was higher (1.65) than for non-CVD mortality (1.34), but not significantly different (p_Diff_=0.067).

To analyse a potential influence of competing risk for CVD mortality, non-CVD mortality was used as the corresponding factor, and vice versa. For model 3 from Table 3 slight but not significant differences per 1 SD of log-transformed hsTnT were observed for CVD mortality with HR=1.56 [1.25–1.96] considering competing risk compared to HR=1.65 [1.32–2.07] (p_Diff_=0.37), and for non-CVD mortality with HR=1.28 [1.13–1.45], compared to HR=1.34 [1.15–1.58] (p_Diff_=0.33).

Since current life expectancy in Germany is 83 for women and 78 years for men, respectively, we analysed premature mortality risk dependent on hsTnT serum levels. In total, 80 participants died before life expectancy was reached (52 women, 28 men). Again, in all models hsTnT was significantly associated with premature mortality (Table 3).

### 3.4 Association of continuous hsTnT values with mortality in participants with and without prevalent CVD

As hsTnT serum concentration was higher in participants with prevalent CVD in contrast to CVD-free persons (**Supplementary Table 5;** delta median = 3.1 ng/L), we compared association between hsTnT and all-cause as well as CVD and non-CVD mortality between those with and without prevalent CVD at baseline (Table 4).

**Table 4:** Cox regression models for association of hsTnT with all-cause, CVD and non-CVD mortality overall and in participants with and without CVD in model 3 (age, sex, eGFR, UACR, diabetes, BMI, physical activity as well as intake of antihypertensive drugs, lipid-lowering medication, and high-ceiling diuretics) with per 1 SD of log-transformed hsTnT values.

Group	All-cause mortality	CVD mortality	Non-CVD mortality
All	385/1670	113/1670	272/1670
	1.36 [1.20–1.55] ***	1.65 [1.32–2.07] ***	1.34 [1.15–1.58] ***
No CVD	111/791	27/791	84/791
	1.28 [0.97–1.69] n.s.	1.47 [0.80–2.73] n.s.	1.31 [0.96–1.79] n.s.
CVD	274/879	86/879	188/879
	1.36 [1.18–1.56] ***	1.65 [1.30–2.10] ***	1.32 [1.10–1.58] **

Numbers of deceased/survivors in groups. HR per 1 SD of log-hsTnT with 95 % CI in square brackets. Significance is marked as * for *p* < 0.05, ** for *p* < 0.01, *** for *p* < 0.001, n.s. not significant (*p* > 0.05).

For participants with prevalent CVD, the effect of hsTnT on all-cause, CVD and non-CVD mortality was significant. In contrast, for CVD-free persons, hsTnT showed no significant association with mortality in the model accounting for age, sex, eGFR, UACR, diabetes, BMI, physical activity as well as intake of antihypertensive drugs, lipid-lowering medication, and high-ceiling diuretics.

### 3.5 Association of continuous hsTnT values with mortality in participants with and without prevalent CAD

As differences in hsTnT values between participants without and with prevalent CAD were observable (**Supplementary Table 5;** delta median = 5.1 ng/L), we restricted the analyses to those with potential myocardial damage from CAD. We compared association between hsTnT and all-cause as well as CVD and non-CVD mortality between those with and without prevalent CAD at baseline (Table 5).

**Table 5:** Cox regression models for association of hsTnT with all-cause, CVD and non-CVD mortality overall and in participants with and without CAD in model 3 (age, sex, eGFR, UACR, diabetes, BMI, physical activity as well as intake of antihypertensive drugs, lipid-lowering medication, and high-ceiling diuretics) with per SD-normalized log-hsTnT values.

Group	All-cause mortality	CVD mortality	Non-CVD mortality
All	385/1670	113/1670	272/1670
	1.36 [1.20–1.55] ***	1.65 [1.32–2.07] ***	1.34 [1.15–1.58] ***
No CAD	278/1453	70/1453	208/1453
	1.30 [1.12–1.52] ***	1.64 [1.22–2.20] **	1.27 [1.07–1.52] **
CAD	107/217	43/217	64/217
	1.47 [1.16–1.85] **	1.52 [1.10–2.10] *	1.66 [1.16–2.36] **

Numbers of deceased/survivors in groups. HR per 1 SD of log-hsTnT with 95 % CI in square brackets. Significance is marked as * for *p* < 0.05, ** for *p* < 0.01, *** for *p* < 0.001, n.s. not significant (*p* > 0.05).

Differences in HR for hsTnT influence on both CVD and non-CVD mortality were not significant (p_Diff_>0.05). hsTnT influence on CVD mortality was slightly but not significantly lower among those with prevalent CAD compared to CAD-free participants (HR=1.52 and HR=1.64, respectively). However, in persons with prevalent CAD, the influence of hsTnT on non-CVD mortality is more pronounced. Therefore, hsTnT influences non-CVD mortality risk in those without prevalent CAD.

In contrast, for the broader spectrum of CVD, slightly higher hsTnT influence on CVD mortality could be observed in those with prevalent CVD compared to the CVD-free group (HR=1.65 and HR=1.47, respectively).

### 3.6 Sex-stratified associations of hsTnT values with mortality

Influence on hsTnT on mortality stratified by sex revealed no substantial changes in HR for all-cause mortality compared to sex-combined analyses (Supplementary Table 6). However, for CVD mortality effect of hsTnT was more pronounced in men compared to women and was not statistically significant in model 3 with CVD adjustment in the female group. This was maybe caused by lower statistical power in women than in men (*n* = 39 and *n* = 74 cases, respectively). No differences for hsTnT influence between men and women were observed for non-CVD mortality. Effect of hsTnT on premature mortality was slightly higher in men compared to women in model 3.

## 4 Discussion

We investigated the relationship between serum levels of high-sensitivity troponin T (hsTnT) and mortality in the general population of older adults. We analysed the association of hsTnT with all-cause, CVD and non-CVD mortality to get an idea for the underlying risk stratification. Even in this cohort of healthier older persons, troponin T was associated with mortality. We found that tertiles of hsTnT showed the tendency of the influence of this marker on mortality, but loses power compared to the continuous biomarker measurement. The assay-specific 99th percentile upper reference limit for hsTnT is 14 ng/L, as recommended in the Universal Definition of Myocardial Infarction and ACC/AHA guidelines [22,23]. Because tertiles are distribution-based and study-specific, we additionally analysed mortality risk using this clinical threshold and compared participants with hsTnT ≥14 ng/L to those with lower baseline values (Supplementary Table 4). Notably, this cut-off lies close to the upper tertile in our cohort (>13.4 ng/L), supporting the clinical relevance of our tertile-based findings.

Unadjusted HR indicated an almost doubling of all-cause mortality risk per 1 SD increase of log-transformed hsTnT (HR = 1.93, 95 % CI = 1.77 – 2.11, *p* = 6.96×10^–47^) and a 2.5-fold increase for CVD mortality risk (HR = 2.51, 95 % CI = 2.14 – 2.94, *p* = 1.05×10^–29^). In addition, we assessed association in persons with and without CAD as a strict definition of prevalent myocardial damage. To extend the analyses to a systemic effect of the marker, we also evaluated hsTnT association with mortality in study participants with and without prevalent CVD, which we defined as CAD, stroke, HF, PAD, or arrhythmia.

As previously reported [4], in our study troponin T also showed association with non-CVD mortality risk. Stratifying for prevalent CVD, we found in the group of participants with CVD 65 % and 32 % increased risk for CVD and non-CVD mortality (per 1 SD of log transformed hsTnT), respectively, after adjusting for age, sex, eGFR, UACR, diabetes, BMI, physical activity as well as intake of antihypertensive drugs, lipid-lowering medication, and high-ceiling diuretics. For participants without CVD at study entry, no significant association with mortality was observed. This was not due a substantially lower sample size in the CVD-free part of the study (902 versus 1153 with CVD; Table 4) but may be driven by the lower mortality rate in the persons without CVD compared to those with CVD at baseline (14.0 % and 31.2 %, respectively). In contrast, restricting the analyses to the more myocardial relevant phenotype of prevalent CAD, we found significant association between hsTnT and both, CVD and non-CVD mortality, in the subgroups of participants with and without CAD (Table 5). Interestingly, the effect was more pronounced for CVD mortality in the CAD-free group compared to participants with prevalent CAD (64 % increased risk per 1 SD of log-transformed hsTnT versus 52 %). The opposite was true for non-CVD mortality: 66 % increased mortality risk per 1 SD of log-transformed hsTnT was observed in the group with prevalent CAD in contrast to 27 % in CAD-free participants. In summary, persons with prevalent CAD showed a high influence of hsTnT on mortality especially for non-CVD causes but also in persons without CAD, mortality risk is higher with elevated hsTnT levels.

The reason why hsTnT not only predicts cardiovascular, but also general and non-cardiovascular mortality might be that increased cardiac load or subclinical cardiac disease, i.e. myocardial hypertrophy, asymptomatic dysfunction, chamber enlargement, valvular dysfunction or rhythm disturbance, which are associated with higher hsTnT impair the ability of the heart to compensate for higher circulatory demands during severe and life-threatening illness such as infection, hypoxia, volume shift, among others.

Interestingly, mortality rates in our study cohort were lower than expected throughout all age strata (Supplementary Table 1). This finding indicates that our study participants, which were all mobile and able to visit the study center, were healthier than the average population. It is noteworthy that hsTnT predicts mortality even in the healthier than average population in the current study.

Troponin T is a biomarker for myocardial damage and necrosis with nearly absolute cardiac specificity [24]. For the diagnosis of acute MI, a measurement exceeding the 99th percentile of a healthy reference population is used; the established cut-off value at the 99th percentile is 14 ng/L troponin in serum [[25], [26], [27], [28]]. Newer assays for troponin T measurements are more sensitive and results in values down to 3 ng/L or even below [3]. Recently, we showed in the AugUR study that a high proportion of the study population without acute coronary syndrome had hsTnT measurements above 14 ng/L and the specificity of this reference limit was only 68 % [5], i.e. 32 % had higher values than 14 ng/L. In this old-aged general population, hsTnT concentrations increased with age even in the population above 70 years [5]. Beyond diagnostic use in emergency department setting, high-sensitivity cardiac troponin measurement together with N-terminal pro-B-type natriuretic peptide (NT-proBNP) was recommended for early detection of heart failure in adults with diabetes [29]. In contrast to cardiac troponin as a marker of cardiomyocyte injury, NT-proBNP is a marker for myocardial stress. In addition, subclinical CVD could be detected in asymptomatic individuals with this biomarkers [30,31]. Cardiac troponins in serum may reflect chronical myocardial injury without clinical manifestation and maybe general atherosclerotic CVD [32].

Our findings are consistent with large community-based cohorts showing that high-sensitivity cardiac troponins predict mortality outside the acute setting. The strength of the association with cardiovascular mortality in AugUR (HR 2.51 unadjusted, 2.17 age–sex adjusted, 1.65 fully adjusted in participants with CVD) closely matches results from the Atherosclerosis Risk in Communities (ARIC), the Age, Gene/Environment Susceptibility–Reykjavik (AGES-Reykjavik) Study, and the Activity and Function in the Elderly in Ulm (ActiFE) Study, where cardiovascular risk increased approximately two- to fourfold across higher troponin strata [[33], [34], [35]]. Effect sizes for all-cause mortality were likewise comparable despite lower absolute mortality rates, consistent with the comparatively healthy and mobile structure of our cohort. By focusing exclusively on individuals aged ≥70 years, our study strengthens evidence that hsTnT retains prognostic value even in very old populations with substantial competing risks. Furthermore, the persistence of associations within strata defined by prevalent cardiovascular disease and coronary artery disease indicates that hsTnT conveys prognostic information beyond clinical disease classification. We also confirm that hsTnT is associated with non-cardiovascular mortality, reinforcing its role as a marker of overall vulnerability rather than a purely disease-specific indicator. A possible explanation is that chronically elevated hsTnT reflects subclinical myocardial injury or structural heart disease that reduces cardiovascular reserve [35,36]. It may also capture broader processes such as vascular aging or multimorbidity not fully reflected by clinical diagnoses, although this cannot be determined from observational data.

From a broader perspective, these findings support the emerging role of high-sensitivity troponin in preventive cardiovascular risk assessment. Prior studies and reviews show that hs-cTn improves prediction beyond traditional risk factors and performs best when incorporated into existing risk models or targeted to higher-risk individuals rather than used for indiscriminate screening [37,38]. Our data extend this concept to very old adults and provide empirical support for future risk models aimed at identifying elderly individuals in whom preventive strategies could be intensified.

### 4.1 Strengths and limitations

We need to acknowledge limitations of our study.

Firstly, AugUR does not represent the general population in the age range from 70 to 95 at baseline visit with an initial net response rate of 18.1 %. In addition, we had a selection for the mobile and healthier older adults because participants had to come to the study centre, and they had to be physically and mentally able to take part in a two-hour examination and interview programme. Comparing to death tables for the whole German population, AugUR study participants showed a substantially lower 5-year mortality rate of 52.6 % in contrast to the expectation in the age-sex-matched general population (Supplementary Table 1). AugUR focuses on general chronic diseases and associated risk factors in persons over 70 years. However, as mentioned above the AugUR population does not fully represent the general population aged 70+. This was therefore reflected by a lower mortality rate than expected for the general population. Because the frailest individuals were less likely to participate, this would be expected to weaken rather than exaggerate associations with mortality, so the observed effects are unlikely to be inflated and may instead be conservative.

Secondly, we have not analysed any echocardiographic and electrocardiographic data or information on cardiomyopathies. Our echocardiographic subcohort is relatively small. In addition, no non-fatal endpoints, e.g. CAD and especially heart failure were analysed.

Thirdly, self-reported heart failure showed low agreement with medical records [15]. CVD is a class of diseases that involve the heart or blood vessels. For our definition of prevalent CVD, we used self-report on coronary artery diseases (MI, stent, bypass) and stroke, as well as peripheral artery disease, heart failure, and measured arrhythmia, but not hypertensive heart disease, rheumatic heart disease, cardiomyopathy, congenital heart disease, valvular heart disease, carditis, aortic aneurysms, thromboembolic disease, and venous thrombosis.

Fourthly, we only included locally recruited people of European descent, and the results can therefore not be generalized.

Fifthly, only hsTnT was analysed and not hsTnI to contrast the different effects on CVD and non-CVD mortality.

Strengths of our study are the long observation period of up to 10 years, complete mortality survey of all study participants and detailed reports on death causes. Our study highlights that hsTnT is a mortality predictor even in the older population over 70 years.

## Consent to publish

Not applicable.

## Availability of data and materials

The datasets generated and analysed during the current study are not publicly available due to data privacy of study participants, but summary data are available from the corresponding authors on reasonable request.

## CRediT authorship contribution statement

**Klaus J. Stark:** Writing – original draft, Validation, Supervision, Project administration, Methodology, Conceptualization. **Martina E. Zimmermann:** Data curation. **Janina M. Herold:** Data curation. **Caroline Brandl:** Investigation. **Ralph Burkhardt:** Resources. **Lars S. Maier:** Writing – review & editing. **Andreas Luchner:** Writing – review & editing, Conceptualization. **Maria A. Heinrich:** Visualization, Data curation. **Iris M. Heid:** Writing – review & editing, Supervision, Conceptualization. **Alexander Dietl:** Writing – original draft, Visualization, Supervision, Project administration, Methodology, Investigation, Formal analysis, Conceptualization.

## Declaration of competing interest

The authors declare the following financial interests/personal relationships which may be considered as potential competing interests:

Iris M. Heid reports financial support was provided by German Research Foundation. Iris M. Heid reports financial support was provided by Roche Diagnostics Deutschland GmbH. Author I.M.H. has received support from Roche Diagnostics Deutschland GmbH for a part of the AugUR study. Roche did not play a role in the study design, in the collection, analysis and interpretation of data, in the writing of the manuscript or in the decision to submit the manuscript for publication. No conflicting relationship exists for the other authorsThe AugUR study was supported by grants from the German Federal Ministry of Education and Research (BMBF 01ER1206 and BMBF 01ER1507) to I.M.H., by the German Research Foundation (DFG HE 3690/7–1 and BR 6028/2–1) to I.M.H. and C.B. and by institutional budget (University of Regensburg). Parts of this work were supported by the German Research Foundation (DFG DI 2876/2–1; 519,332,007 to A.D. Roche Diagnostics Deutschland GmbH has provided kits for assessment of hsTnT free of charge for a part of the study (Roche contract number 405,196), but it did not play a role in the study design, in the collection, analysis, and interpretation of data, in the writing of the manuscript or in the decision to submit the manuscript for publication.

If there are other authors, they declare that they have no known competing financial interests or personal relationships that could have appeared to influence the work reported in this paper.

## Appendix Supplementary materials

Image, application 1
