# Supplementary material for: Association of high-sensitivity cardiac troponin T with all-cause and cardiovascular mortality in a mobile cohort of older adults aged 70 to 95 years – Results from the AugUR study
Source: Am J Prev Cardiol. 2026 Mar 3;27:101520. doi: 10.1016/j.ajpc.2026.101520 (PMC13261262; doi:10.1016/j.ajpc.2026.101520)
Supplement: Supplementary file 1 [file mmc1.docx]

# Supplementary information

Recruitment scheme of the AugUR study for the baseline recruitment from 2013 to 2019 is shown in **Supplementary Figure 1** together with data on mortality in the follow-up.


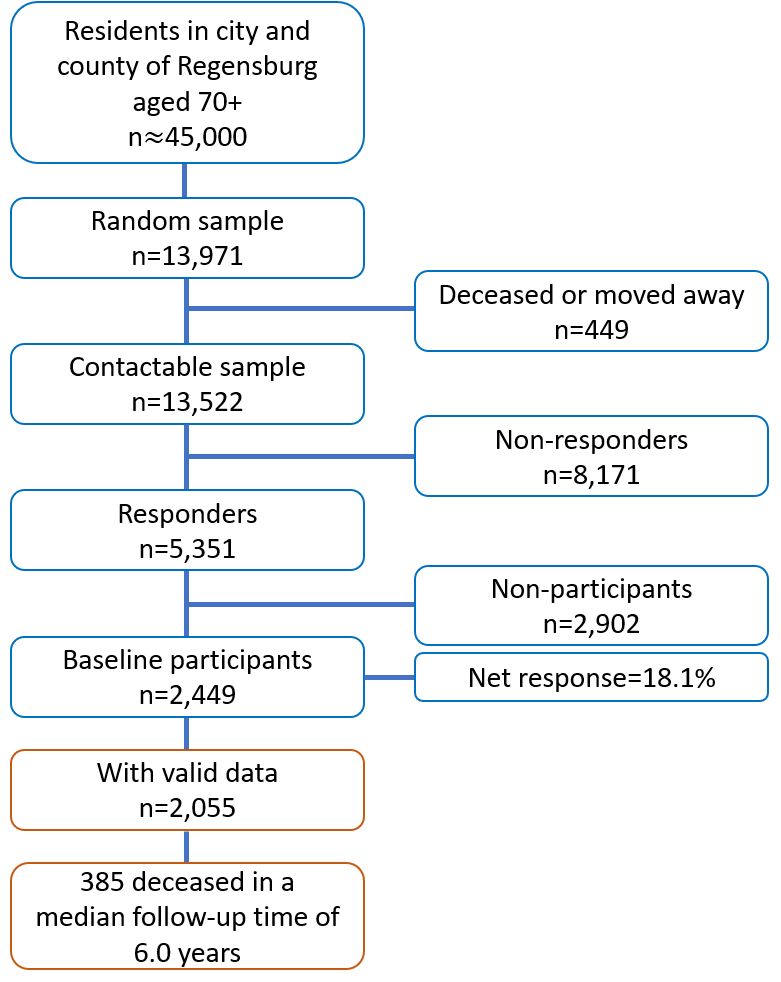


**Supplementary Figure 1. Overview of AugUR study recruitment.** Given are number of participants at baseline in general and with valid hsTnT data and information on mortality in the follow-up used in the present analyses (red).

**Comparison of 5-year mortality between population and study.**

To understand the study population, the question was to answer, to what extend the mortality rate of the AugUR study population of mobile elderly differs from the general population of the same age. The comparison between mortality rate of AugUR study participants and the general German population is shown in **Supplementary Table 1**.

**Supplementary Table 1.** Age- and sex-groups in AugUR with expected and observed mortality rate from German death tables 2019/2021.

| Sex-age-group | Probability of death after 5 years* | Number in AugUR study | Expected deaths after 5 years | Observed deaths after 5 years | Ratio  observed/  expected |
| --- | --- | --- | --- | --- | --- |
| m70-74 | 12.65% | 301 | 38.1 | 30 | 78.7% |
| m75-89 | 18.99% | 363 | 68.9 | 35 | 50.8% |
| m80-84 | 30.51% | 202 | 61.6 | 25 | 40.6% |
| m85-89 | 50.20% | 93 | 46.7 | 30 | 64.2% |
| m90-94 | 72.06% | 22 | 15.9 | 10 | 62.9% |
| f70-74 | 7.58% | 303 | 23.0 | 11 | 47.8% |
| f75-89 | 12.07% | 410 | 49.5 | 29 | 58.6% |
| f80-84 | 21.70% | 247 | 53.6 | 24 | 44.8% |
| f85-89 | 40.55% | 94 | 38.1 | 12 | 31.5% |
| f90-94 | 64.54% | 18 | 11.6 | 7 | 60.3% |
| f95-99 | 83.43% | 2 | 1.7 | 2 | 117.6% |
| Total |  | 2,055 | 409 | 215 | 52.6% |

* From German death tables 2019/2021 (www.destatis.de; 12621-04). m, male; f, female. Age in years.

Referring to the official German life expectancy and death tables 2019/2021 (www.destatis.de; 12621-04) for the general population stratified for men and women aged 70 to 95 years, expected 5-year mortality rate would result in 409 deceased persons from the 2,055 study participants. Explicitly, the “healthy” status of AugUR study participants can be seen by the reduced 5-year mortality rate with 215 events from the expectation of 409 deaths in the age-sex-matched general population (52.6%).

**Association with CVD mortality**

To assess for association of different factors with CVD mortality, Cox proportional hazard survival regression was applied with -2-log-liklehood-ratio forward selection with baseline variables from **Table 1**.

Higher age, UACR and hsTnT as well as CAD and intake of high-ceiling diuretics (as a marker of severe cardiovascular disturbances) increased the risk for CVD mortality. Lipid-lowering medication, higher BMI and physical activity decreased the risk. Whether the study participants came from the AugUR1 or AugUR2 recruitment wave had no influence on mortality despite the different long observation periods. CAD but not CVD was integrated in the model as a significant predictor for CVD mortality (**Supplementary Table 2**).

**Supplementary Table 2.** Significant factors influencing CVD mortality risk in Cox proportional hazard regression using forward selection.

|  | HR | 95% CI | p-value |
| --- | --- | --- | --- |
| hsTnT, per SD on log-scale | 1.74 | 1.43-2.11 | 2.59*10^-8^ |
| CAD | 2.94 | 1.87-4.58 | 2.70*10^-6^ |
| Age [yrs] | 1.08 | 1.05-1.12 | 1.02*10^-5^ |
| UACR on log-scale | 1.31 | 1.14-1.50 | 6.56*10^-5^ |
| Physical activity | 0.47 | 0.31-0.70 | 2.56*10^-4^ |
| Lipid-lowering medication | 0.51 | 0.33-0.78 | 2.02*10^-3^ |
| BMI [kg/m²] | 0.94 | 0.89-0.98 | 6.56*10^-3^ |
| Intake of high-ceiling diuretics | 1.68 | 1.06-2.65 | 0.027 |

HR, hazard ratio, CI, confidence interval, SD, standard deviation, CAD, coronary artery disease, UACR, urinary creatinine-to-albumin ratio, BMI, body mass index.

**Association with hsTnT serum levels**

Age, sex, eGFR, UACR, diabetes, CVD (but not CAD), physical activity, intake of antihypertensive drugs, high-ceiling diuretics and lipid-lowering medication showed an effect on hsTnT serum levels (**Supplementary Table 3**) in forward selection applying linear regression.

**Supplementary Table 3.** Significant factors influencing hsTnT serum levels (per SD on log-scale) in linear regression using forward selection.

|  | beta | 95% CI | p-value |
| --- | --- | --- | --- |
| Sex (male) | 0.618 | 0.553-0.684 | 5.45*10^-70^ |
| eGFR | -0.017 | (-0.019)-(-0.015) | 3.63*10^-46^ |
| Age [yrs] | 0.044 | 0.037-0.051 | 8.26*10^-33^ |
| UACR on log-scale | 0.116 | 0.088-0.144 | 7.15*10^-16^ |
| High-ceiling diuretics | 0.359 | 0.251-0.466 | 8.28*10^-11^ |
| CVD | 0.125 | 0.055-0.195 | 4.87*10^-4^ |
| Antihypertensive drugs | 0.110 | 0.035-0.186 | 4.26*10^-3^ |
| BMI [kg/m²] | 0.936 | 0.892-0.982 | 6.56*10^-3^ |
| Physical activity | -0.110 | (-0.194)-(-0.026) | 0.010 |
| Diabetes | 0.100 | 0.016-0.185 | 0.020 |
| Lipid-lowering medication | -0.079 | (-0.152)-(-0.006) | 0.034 |

CI, confidence interval, eGFR, estimated glomerular filtration rate, CVD, cardiovascular disease, BMI, body mass index.

**Supplementary Table 4.** Hazard ratios from Cox proportional hazard survival regression for mortality by clinical cut-off for hsTnT distribution (14 ng/L).

| # for mortality analyses:  All-cause/CVD/non-CVD | hsTnT <14 ng/L  (n=1437/1294/1397) | hsTnT ≥14 ng/L  (n=618/489/545) |
| --- | --- | --- |
| All-cause mortality | 183 (12.7) | 202 (32.7) |
| Unadjusted | Reference | 3.13 [2.56;3.82] *** |
| Model 1 | Reference | 2.00 [1.59;2.51] *** |
| Model 2 | Reference | 1.46 [1.14;1.87] ** |
| Model 3 | Reference | 1.40 [1.09;1.80] ** |
| Model 3+CAD | Reference | 1.38 [1.07;1.78] * |
| Model 3+CVD | Reference | 1.37 [1.07;1.77] * |
| CVD mortality | 40 (3.1) | 73 (14.9) |
| Unadjusted | Reference | 5.75 [3.91;8.46] *** |
| Model 1 | Reference | 3.41 [2.20;5.29] *** |
| Model 2 | Reference | 2.25 [1.41;3.60] *** |
| Model 3 | Reference | 2.16 [1.35;3.47] ** |
| Model 3+CAD | Reference | 2.15 [1.32;3.50] ** |
| Model 3+CVD | Reference | 2.11 [1.31;3.39] ** |
| Non-CVD mortality | 143 (10.2) | 129 (23.7) |
| Unadjusted | Reference | 2.72 [2.14;3.45] *** |
| Model 1 | Reference | 1.75 [1.34;2.29] *** |
| Model 2 | Reference | 1.29 [0.96;1.72] n.s. |
| Model 3 | Reference | 1.26 [0.94;1.70] n.s. |
| Model 3+CAD | Reference | 1.24 [0.92;1.67] n.s. |
| Model 3+CVD | Reference | 1.23 [0.91;1.65] n.s. |

Given are HR and [95% CI] for hsTnT ≥ 14 ng/L compared to the group with hsTnT < 14 ng/L as reference for all-cause, CVD and non-CVD mortality (number and percentage of deceased are given). Significance is marked as * for p<0.05, ** for p<0.01, *** for p<0.001, n.s. not significant (p>0.05). Note: one model per line with hsTnT groups as one categorial variable was applied. Unadjusted: hsTnT group ≥14 ng/L compared to < 14 ng/L; Model 1: including age and sex; Model 2: additionally eGFR_crea-cys_ and UACR (on log-scale); Model 3: additionally diabetes, BMI, physical activity as well as intake of antihypertensive drugs, lipid-lowering medication and high-ceiling diuretics; Model 3+CAD: as model 3 with CAD; Model 3+CVD: as model 3 with CVD.

In a separate analysis with the model from Supplementary Table 3 both, prevalent CVD and CAD showed an influence on hsTnT levels. Interestingly, CVD had a pronounced effect on hsTnT compared to CAD in the full model (b=0.125 versus 0.073).

In conclusion, for association between hsTnT levels and mortality, age, sex, eGFR, UACR, diabetes, BMI, physical activity as well as intake of antihypertensive drugs, lipid-lowering medication, and high-ceiling diuretics should be considered. Due to the different effects of CAD and CVD, respectively, on both hsTnT levels and mortality, these two disease entities must be considered separately (**Supplementary Table 5**).

**Supplementary Table 5.** Baseline characteristics and mortality data of AugUR participants overall and by **prevalent CVD and CAD status**.

| Characteristic | Overall  (n=2,055) | No CVD  (n=902) | CVD  (n=1,153) | *p-value*  *No CVD/CVD* | No CAD  (n=1,731) | CAD  (n=324) | *p-value*  *No CAD/CAD* |
| --- | --- | --- | --- | --- | --- | --- | --- |
| *General descriptives* |  |  |  |  |  |  |  |
| Age [yrs] | 78.3 ± 5.0 | 77.3 ± 4.6 | 79.0 ± 5.1 | *3.04*10^-15^* | 78.1 ± 4.9 | 79.5 ± 5.2 | *3.46*10^-6^* |
| Age range [yrs] | 70.3 – 95.0 | 70.3 – 95.0 | 70.4 – 95.0 |  | 70.3 – 95.0 | 70.4 – 95.0 |  |
| Sex, men | 981 (47.7) | 368 (40.8) | 613 (53.2) | *2.55*10^-8^* | 747 (43.2) | 234 (72.2) | *6.98*10^-22^* |
| Never smoked | 1,129 (54.9) | 527 (58.4) | 602 (52.2) | *4.96*10^-3^* | 998 (57.7) | 131 (40.4) | *1.08*10^-8^* |
| Smoked within last 15 yrs | 219 (10.7) | 84 (9.3) | 135 (11.7) | *0.081* | 176 (10.2) | 43 (13.3) | *0.097* |
| Grip strength [kg] | 30.2 ± 9.8 | 30.1 ± 9.8 | 30.3 ± 9.8 | *0.633* | 29.8 ± 9.8 | 32.6 ± 9.4 | *3.15*10^-6^* |
| Physical activity ≥2 hrs/week | 1,658 (80.7) | 776 (86.0) | 882 (76.5) | *5.54*10^-8^* | 1,419 (82.0) | 239 (73.8) | *5.91*10^-4^* |
| BMI [kg/m²] | 27.7 ± 4.5 | 27.3 ± 4.4 | 28.0 ± 4.5 | *4.65*10^-4^* | 27.5 ± 4.5 | 28.3 ± 4.2 | *4.59*10^-3^* |
| WHR | 0.95 ± 0.09 | 0.94 ± 0.09 | 0.96 ± 0.09 | *1.95*10^-7^* | 0.94 ± 0.09 | 0.99 ± 0.09 | *4.84*10^-20^* |
| Systolic BP [mmHg] | 131.8 ± 18.0 | 133.2 ± 17.0 | 130.9 ± 18.7 | *2.19*10^-3^* | 132.4 ± 18.0 | 128.9 ± 17.7 | *1.43*10^-3^* |
| Diastolic BP [mmHg] | 76.2 ± 10.6 | 77.8 ± 9.9 | 74.9 ± 11.0 | *8.91*10^-10^* | 76.9 ± 10.5 | 72.2 ± 10.4 | *2.65*10^-13^* |
| Pulse frequency [min^-1^] | 69.4 ± 11.5 | 70.1 ± 10.8 | 68.9 ± 11.9 | *0.018* | 70.3 ± 11.3 | 65.0 ± 11.1 | *2.13*10^-14^* |
| *Medication* |  |  |  |  |  |  |  |
| Antihypertensive drugs | 1378 (67.1) | 510 (56.5) | 868 (75.3) | *2.96*10^-19^* | 1,088 (62.9) | 290 (89.5) | *7.40*10^-21^* |
| High-ceiling diuretics | 267 (13.0) | 47 (5.2) | 220 (19.1) | *1.69*10^-20^* | 162 (9.4) | 105 (32.4) | *9.88*10^-30^* |
| Lipid-lowering | 725 (35.3) | 219 (24.3) | 506 (43.9) | *2.70*10^-20^* | 480 (27.7) | 245 (75.6) | *1.45*10^-61^* |
| Antidiabetics | 339 (16.5) | 108 (12.0) | 231 (20.0) | *1.03*10^-6^* | 243 (14.0) | 96 (29.6) | *3.92*10^-121^* |

**Supplementary Table 5 continued.**

| Characteristic | Overall  (n=2,055) | No CVD  (n=902) | CVD  (n=1,153) | *p-value*  *No CVD/CVD* | No CAD  (n=1,731) | CAD  (n=324) | *p-value*  *No CAD/CAD* |
| --- | --- | --- | --- | --- | --- | --- | --- |
| *Laboratory values* |  |  |  |  |  |  |  |
| hsTnT [ng/L] | 10.4 [7.2;15.3] | 9.1 [6.5;12.3] | 12.2 [8.1;18.5] | *1.64*10^-31^* | 9.9 [7.0;14.2] | 15.0 [10.1;20.7] | *4.95*10^-26^* |
| hsTnT, per SD on log scale | 3.97 ± 1.00 | 3.68 ± 0.85 | 4.20 ± 1.05 | *3.27*10^-34^* | 3.87 ± 0.96 | 4.51 ± 1.05 | *2.43*10^-22^* |
| Min – Max | 1.83 – 7.68 | 1.83 – 6.56 | 1.83 – 7.68 |  | 1.83 – 7.68 | 1.83 – 7.68 |  |
| eGFR [ml/min/1.73m²] | 69.3 ± 17.1 | 73.3 ± 15.5 | 66.2 ± 17.6 | *1.18*10^-21^* | 71.0 ± 16.4 | 60.6 ± 18.0 | *4.91*10^-20^* |
| UACR [mg/g] | 10.0 [5.1;22.6] | 8.3 [4.5;16.2] | 11.8 [5.7;29.0] | *7.79*10^-13^* | 9.7 [5.0;21.0] | 13.0 [5.8;36.1] | *3.27*10^-5^* |
| LDL cholesterol [mg/dl] | 141.1 ± 34.9 | 148.9 ± 31.4 | 134.9 ± 36.2 | *1.31*10^-20^* | 145.2 ± 33.4 | 118.7 ± 34.4 | *9.91*10^-38^* |
| HDL cholesterol [mg/dl] | 61.4 ± 15.5 | 63.7 ± 14.9 | 59.6 ± 15.6 | *1.96*10^-9^* | 63.0 ± 15.4 | 52.8 ± 12.8 | *9.35*10^-33^* |
| Total cholesterol [mg/dl] | 217.7 ± 45.9 | 229.0 ± 40.8 | 208.9 ± 47.7 | *2.51*10^-24^* | 224.0 ± 43.7 | 183.7 ± 42.2 | *1.53*10^-50^* |
| Triglycerides [mg/dl] | 137 [99;189] | 140 [100;189] | 136 [98;189] | *0.369* | 137 [98;187] | 144 [105;199] | *0.050* |
| HbA1c [%] | 5.8 ± 0.7 | 5.7 ± 0.6 | 5.9 ± 0.7 | *1.79*10^-7^* | 5.7 ± 0.7 | 6.0 ± 0.7 | *1.38*10^-11^* |
| CRP [mg/l] | 0.3 [0.3;0.4] | 0.3 [0.3;0.4] | 0.3 [0.3;0.5] | *3.61*10^-4^* | 0.3 [0.3;0.4] | 0.3 [0.3;0.5] | *0.071* |
|  |  |  |  |  |  |  |  |
| *Diseases* |  |  |  |  |  |  |  |
| Diabetes mellitus | 434 (21.1) | 138 (15.3) | 296 (25.7) | *1.08*10^-8^* | 313 (18.1) | 121 (37.3) | *6.34*10^-15^* |
| Hypertension | 1,495 (72.7) | 602 (66.7) | 893 (77.5) | *6.23*10^-8^* | 1,222 (70.6) | 273 (86.3) | *3.98*10^-7^* |
| CKD | 572 (27.8) | 178 (19.7) | 394 (34.2) | *4.26*10^-13^* | 422 (24.4) | 150 (46.3) | *6.54*10^-16^* |
| Microalbuminuria | 360 (17.5) | 122 (13.5) | 238 (20.6) | *5.49*10^-6^* | 287 (16.6) | 73 (22.5) | *2.22*10^-3^* |
| Macroalbuminuria | 56 (2.7) | 9 (1.0) | 47 (4.1) | *4.82*10^-6^* | 33 (1.9) | 23 (7.1) | *1.83*10^-8^* |

**Supplementary Table 5 continued.**

| Characteristic | Overall  (n=2,055) | No CVD  (n=902) | CVD  (n=1,153) | *p-value*  *No CVD/CVD* | No CAD  (n=1,731) | CAD  (n=324) | *p-value*  *No CAD/CAD* |
| --- | --- | --- | --- | --- | --- | --- | --- |
| *Mortality data* |  |  |  |  |  |  |  |
| Follow-up time | 6.0 [4.6;8.3] | 6.1 [4.8;8.5] | 5.9 [4.5;8.2] | *2.09*10^-6^* | 6.0 [4.7;8.4] | 5.9 [4.4;8.2] | *0.053* |
| Survivors | 1,670 (81.3) | 791 (87.7) | 879 (76.2) | *3.95*10^-11^* | 1,453 (83.9) | 217 (67.0) | *6.84*10^-13^* |
| Follow-up time | 6.1 [4.8;8.5] | 6.2 [4.9;8.6] | 6.0 [4.8;8.5] | *0.022* | 6.1 [4.8;8.5] | 6.2 [4.9;8.7] | *0.666* |
| hsTnT, per SD on log scale | 3.8 ± 0.9 | 3.6 ± 0.8 | 4.0 ± 1.0 | *8.93*10^-14^* | 3.8 ± 0.9 | 4.3 ± 1.0 | *2.49*10^-21^* |
| Min - Max | 1.83 – 7.68 | 1.83 – 6.45 | 1.83 – 7.68 |  | 1.83 – 7.68 | 1.83 – 7.39 |  |
| All-cause mortality | 385 (18.7) | 111 (12.3) | 274 (23.8) | *3.95*10^-11^* | 278 (16.1) | 107 (33.0) | *6.84*10^-13^* |
| Follow-up time deceased | 4.6 [2.9;6.8] | 4.9 [3.3;7.3] | 4.2 [2.7;6.5] | *0.055* | 4.6 [2.9;6.9] | 4.6 [2.6;6.5] | *0.775* |
| Deceased men | 245 (63.6) | 63 (56.8) | 182 (66.4) | *0.074* | 163 (58.6) | 82 (76.6) | *1.00*10^-3^* |
| hsTnT, per SD on log scale | 4.5 ± 1.1 | 4.1 ± 0.9 | 4.7 ± 1.1 | *3.16*10^-7^* | 4.4 ± 1.1 | 5.0 ± 1.0 | *1.07*10^-7^* |
| Min - Max | 1.83 – 7.68 | 1.83 – 6.56 | 1.83 – 7.68 |  | 1.83 – 7.68 | 1.83 – 7.68 |  |
| CVD mortality | 113 (6.3) | 27 (3.3) | 86 (8.9) | *1.26*10^-6^* | 70 (4.6) | 43 (16.5) | *2.78*10^-13^* |
| Follow-up time deceased | 5.2 [3.3;7.3] | 6.1 [4.1;8.5] | 4.6 [3.0;7.0] | *0.013* | 5.4 [3.5;7.5] | 5.0 [2.5;7.0] | *0.206* |
| Deceased men | 74 (65.5) | 15 (55.6) | 59 (68.6) | *0.213* | 41 (58.6) | 33 (76.7) | *0.049* |
| hsTnT, per SD on log scale | 4.8 ± 1.1 | 4.3 ± 1.0 | 4.9 ± 1.1 | *0.011* | 4.6 ± 1.1 | 5.1 ± 1.2 | *0.016* |
| Min - Max | 1.83 – 7.68 | 1.83 – 5.99 | 1.83 – 7.68 |  | 1.83 – 7.68 | 1.83 – 7.68 |  |
| Non-CVD mortality | 272 (14.0) | 84 (9.6) | 188 (17.6) | *4.05*10^-7^* | 208 (12.5) | 64 (22.8) | *4.64*10^-6^* |
| Follow-up time deceased | 4.4 [2.7;6.6] | 4.7 [3.0;6.9] | 4.2 [2.6;6.4] | *0.286* | 4.2 [2.7;6.6] | 4.5 [2.6;6.2] | *0.818* |
| Deceased men | 171 (62.9) | 48 (57.1) | 123 (65.4) | *0.191* | 122 (57.7) | 49 (76.6) | *0.010* |
| hsTnT, per SD on log scale | 4.4 ± 1.1 | 4.1 ± 0.9 | 4.6 ± 1.1 | *1.28*10^-5^* | 4.3 ± 1.1 | 4.9 ± 0.9 | *2.89*10^-5^* |
| Min - Max | 1.83 – 7.68 | 1.83 – 6.56 | 1.83 – 7.68 |  | 1.83 – 7.68 | 2.87 – 7.21 |  |

Continuous values are means ± standard deviation if normally distributed; other continuous values are median with 25%^th^; 75%^th^ quartiles in square brackets; categorial variables are total numbers and percent in brackets.

BMI, body mass index; WHR, waist-hip ratio; BP blood pressure; hsTnT, high-sensitivity cardiac troponin T; eGFR, estimated glomerular filtration rate calculated using serum creatinine and cystatin C applying the CKD-Epi formula 2021 (20); UACR, urinary albumin-to-creatinine ratio; LDL, low-density lipoprotein; HDL, high-density lipoprotein; HbA1c, glycated haemoglobin A1c; CRP, C-reactive protein; CKD, chronic kidney disease defined as eGFR < 60 ml/min/1.73m²; microalbuminuria defined UACR 30-300 mg/g; macroalbuminuria defined as UACR > 300 mg/g; CAD, coronary artery disease defined as self-reported history of myocardial infarction and/or percutaneous coronary intervention and/or coronary bypass surgery; CVD, defined as CAD or stroke or HF (self-reported heart failure, “heart weakness”) or arrhythmia (defined as measured irregular pulse at study centre) or PAD (peripheral artery disease). For CVD mortality, non-CVD death was set as missing and vice versa. Two-sided p-values for comparison between individuals without and with prevalent CVD or CAD were derived from T-test for normal distributed continuous variables, from Mann-Whitney-U-test for not normal distributed continuous variables, and from Chi²-test for nominal variables.

**Supplementary Table 6.** Cox regression models for association between per 1 SD of log-transformed hsTnT and mortality stratified by sex.

|  | All-cause mortality | CVD mortality | Non-CVD mortality | Premature mortality |
| --- | --- | --- | --- | --- |
| Women | n=140/934 | n=39/934 | n=101/934 | n=52/934 |
| Unadjusted | 1.91 [1.64-2.22] *** | 2.31 [1.72-3.09] *** | 1.88 [1.57-2.25] *** | 1.58 [1.22-2.06] *** |
| Model 1 | 1.65 [1.39-1.97] *** | 1.83 [1.31-2.56] *** | 1.66 [1.35-2.04] *** | 2.04 [1.58-2.63] *** |
| Model 2 | 1.40 [1.15-1.69] *** | 1.51 [1.03-2.22] * | 1.41 [1.13-1.77] ** | 1.62 [1.20-2.19] ** |
| Model 3 | 1.32 [1.09-1.60] ** | 1.41 [0.97-2.06] n.s. | 1.33 [1.06-1.66] * | 1.62 [1.20-2.20] ** |
| Model 3+CAD | 1.31 [1.08-1.59] ** | 1.46 [1.01-2.12] * | 1.33 [1.06-1.66] * | 1.62 [1.20-2.19] ** |
| Model 3+CVD | 1.29 [1.07-1.57] ** | 1.39 [0.95-2.02] n.s. | 1.31 [1.04-1.64] * | 1.60 [1.18-2.17] ** |
| Men | n=245/736 | n=74/736 | n=171/736 | n=28/736 |
| Unadjusted | 1.87 [1.66-2.11] *** | 2.54 [2.01-3.11] *** | 1.81 [1.55-2.12] *** | 1.63 [1.12-2.40] * |
| Model 1 | 1.66 [1.45-1.90] *** | 2.37 [1.88-2.99] *** | 1.57 [1.32-1.87] *** | 2.03 [1.44-2.86] *** |
| Model 2 | 1.44 [1.22-1.69] *** | 1.95 [1.49-2.56] *** | 1.36 [1.11-1.67] ** | 1.86 [1.24-2.78] ** |
| Model 3 | 1.38 [1.17-1.63] *** | 1.85 [1.39-2.46] *** | 1.34 [1.08-1.66] ** | 1.88 [1.23-2.88] ** |
| Model 3+CAD | 1.36 [1.15-1.61] *** | 1.75 [1.32-2.32] *** | 1.34 [1.08-1.65] ** | 1.86 [1.21-2.86] ** |
| Model 3+CVD | 1.36 [1.15-1.61] *** | 1.81 [1.36-2.41] *** | 1.32 [1.07-1.64] ** | 1.82 [1.19-2.77] ** |
|  |  |  |  |  |

Given are HR and [95% CI] per 1 SD of log-hsTnT for all-cause, CVD and non-CVD mortality. Significance is marked as * for p<0.05, ** for p<0.01, *** for p<0.001, n.s. not significant (p>0.05). Unadjusted: SD-normalized log-scaled hsTnT, continuous; Model 1: including age; Model 2: additionally eGFR_crea-cys_ and UACR (on log-scale); Model 3: additionally diabetes, BMI, physical activity as well as intake of antihypertensive drugs, lipid-lowering medication and high-ceiling diuretics; Model 3+CAD: as model 3 with CAD; Model 3+CVD: as model 3 with CVD.
